# Three new species of *Junghuhnia* (Polyporales, Basidiomycota) from China

**DOI:** 10.3897/mycokeys.72.51872

**Published:** 2020-08-14

**Authors:** Ping Du, Fang Wu, Xue-Mei Tian

**Affiliations:** 1 College of Life Science and Technology, Yangtze Normal University, Chongqing 408100, China Yangtze Normal University Chongqing China; 2 School of Ecology and Nature Conservation, Institute of Microbiology, Beijing Forestry University, Beijing 100083, China Beijing Forestry University Beijing China; 3 Shandong Provincial Key Laboratory of Applied Mycology, Qingdao Agricultural University, Qingdao 266109, China Qingdao Agricultural University Qingdao China

**Keywords:** Steccherinaceae, polypore, wood-inhabiting fungi

## Abstract

In this study, taxonomic and phylogenetic analyses of *Junghuhnia* were performed. Three new species were characterised according to morphological characteristics and molecular phylogenetic analysis using ITS and nLSU sequences. They are *J. austrosinensis***sp. nov.**, *J. nandinae***sp. nov.** and *J. subcollabens***sp. nov.***Junghuhnia austrosinensis* is characterised by resupinate, thin basidiomata with white to buff-yellow hymenophore, small pores (9–11 per mm), clamped generative hyphae possessing hymenial cystidia, ellipsoid basidiospores (2.5–3 × 1.7–2 µm) and growth on fallen bamboo or angiosperm branch. *Junghuhnia nandinae* is characterised by resupinate basidiomata with pink to salmon pores and a distinct white margin, clamp generative hyphae, interwoven tramal hyphae, ellipsoid basidiospores measuring 2.6–3.2 × 1.8–2 µm and growth on *Nandina domestica*. *Junghuhnia subcollabens* is characterised by resupinate basidiomata with pale salmon to brownish vinaceous hymenophore, small pores (10–12 per mm), generative hyphae with simple septa and clamp connections, interwoven tramal hyphae, lunate basidiospores measuring 2.9–3.4 × 1.6–1.8 µm and thriving on rotten wood of angiosperms.

## Introduction

Corda established the genus *Junghuhnia* Corda emend. Ryvarden on the type *Laschia crustacea* Jungh. *Junghuhnia* is characterised by a dimitic hyphal system with clamped generative hyphae and cyanophilous skeletal hyphae, smooth or encrusted skeletocystidia and subglobose or cylindrical basidiospores (Ryvarden and Gilbertson 1993; Núñez and Ryvarden 2001; Yuan and Dai 2008; Yuan et al. 2012). *Junghuhnia* is polyphyletic and has a complicated phylogenetic relationship with *Antrodiella* Ryvarden & I. Johans. and *Steccherinum* Gray (Miettinen et al. 2012; Westphalen et al. 2018; Yuan et al. 2019). These three genera share dimitic hyphal structure with cyanophilous skeletal hyphae and small, smooth, inamyloid, acyanophilous basidiospores (Dai et al. 2004). *Junghuhnia* and *Antrodiella* have poroid hymenophores, while *Steccherinum* have hydnaceous to odontioid hymenophores and *Junghuhnia* differs from *Antrodiella* by having skeletocystidia (Yuan et al. 2012). Previously, more than 30 species were accepted in the genus (Yuan et al. 2012, 2019; Ryvarden 2018, 2019) and 16 species were recorded in China (Yuan and Dai 2008; Miettinen et al. 2012; Yuan et al. 2012, 2019; Wu et al. 2020).

During recent studies on wood-inhabiting fungi in China, samples morphologically belonging to *Junghuhnia* were collected. After microscopic examination and phylogenetic analysis of ITS and nLSU sequences, we identified three new lineages in *Junghuhnia* and they are different from the existing fungal taxa. Therefore, three novel *Junghuhnia* species are characterised.

## Materials and methods

### Morphology

The samples were evaluated and submitted at the Institute of Microbiology herbaria of BJFC (Beijing Forestry University) and IFP (Institute of Applied Ecology, Chinese Academy of Sciences). The field notes formed the basis of macro-morphological details. Microscopic examination (magnifications ≤ 1000×; Nikon Eclipse 80i microscope) of the sections in phase contrast illumination was undertaken as per the protocols by Dai (2010) and Cui et al. (2019). A drawing tube was used to prepare the drawings. The sections were stained using Melzer’s reagent and Cotton Blue to carry out measurements, assess microscopic features and prepare drawings. Sections from the tubes were used to assess the spores. To show the variation in spore sizes, from both ends of the range, 5% of measurements were excluded and are mentioned in parentheses. Abbreviations include KOH, potassium hydroxide (5%); IKI–, Melzer’s reagent negative; IKI, Melzer’s reagent; CB+, cyanophilous in Cotton blue; Q, the L/W ratio; W, mean spore width and L, mean spore length (both L and W: arithmetic average of all spores); n = number of spores in a specified number of specimens. The terms used for special colour are as per Rayner (1970) and Petersen (1996).

### Molecular phylogenetic study

Genomic DNA was isolated from the dried specimens using the CTAB rapid plant genome extraction kit from Aidlab Biotechnologies (Beijing, China), as per provided guidelines with few alterations. The ITS5 and ITS4 primers were used (White et al. 1990) for the amplification of ITS sequences through PCR and the LR0R and LR7 primers were used for nLSU (Vilgalys and Hester 1990). The PCR process for ITS was: 95 °C for 3 min for initial denaturation; 35 cycles for 40 sec at 94 °C, 45 sec at 54 °C, 1 min at 72 °C, 72 °C for 10 min (final extension). The PCR process for nLSU was: 94 °C for 1 min for initial denaturation, 35 cycles for 1 min at 94 °C, 1 min at 50 °C, 1.5 min at 72 °C and 72 °C for 10 min (final extension). After purification of the products from PCR, they were sequenced at Beijing Genomics Institute (China) using the same set of primers.

Phylogenetic analyses were applied to the combined ITS+nLSU dataset. Sequences generated in this study were aligned with additional sequences downloaded from GenBank (Table 1) referred to Miettinen et al. (2012) and Yuan et al. (2019). The alignment of the dataset with *Exidiopsis calcea* (Pers.) K. Wells, as the outgroup following Yuan et al. (2016), was done applying MAFFT 7 with the option of G-INS-i (Katoh and Standley 2013) and the outcome was deposited at TreeBase (submission ID 25589). Construction of the ML (Maximum Likelihood) tree was done applying raxmlGUI 1.2 (Stamatakis 2006; Silvestro and Michalak 2012) with the model GTR + I + G and the option of auto FC (Pattengale 2010) in BS (bootstrap) replicates. The determination of the best-fit evolution model was done using MrModeltest2.3 (Posada and Crandall 1998; Nylander 2004) for the combined dataset for estimating BI (Bayesian Inference), which was estimated using MrBayes3.2.5 (Ronquist et al. 2012). From random starting trees, two runs of four Markov chains were run for the combined datasets for 1 million generations and, every 100 generations, trees were sampled. The initial generations (one-fourth) were rejected as burn-in. Then, for all remaining trees, the majority rule consensus tree was calculated. Branches were considered as significantly supported if they received bootstrap support (BS) for Bayesian posterior probabilities (BPP) and Maximum Likelihood ≥ 0.95 (BPP) and 75% (BS), respectively.

**Table 1.** Information for the sequences used in this study.

Species	Specimen no.	Locality	GenBank accession no.	
			ITS	nLSU
*Antrodiella americana*	HHB 4100-Sp	United States	EU232186	EU232270
*Antrodiella faginea*	KH Larsson 11977	Sweden	JN710514	JN710514
*Antrodiella foliaceodentata*	LE 247382	Russia	JN710515	JN710515
*Antrodiella onychoides*	Miettinen 2312	Finland	JN710517	JN710517
*Antrodiella pallescens*	Miettinen X1080	Sweden	JN710518	JN710518
*Antrodiella romellii*	Miettinen 7429	Finland	JN710520	JN710520
*Antrodiella semisupina*	Labrecque & Labbé 372	Canada	JN710521	JN710521
*Ceriporiopsis aneirina*	MUAF 888	Czech Republic	EU340895	EU368503
*Ceriporiopsis balaenae*	Niemelä 2752	Canada	FJ496669	FJ496717
*Exidiopsis calcea*	MW 331	Canada	AF291280	AF291326
*Frantisekia mentschulensis*	BRNM 710170	Czech Republic	FJ496670	FJ496728
*Frantisekia abieticola*	Cui10525	China	KC485534	KC485552
*Gloeoporus citrinoalbus*	Yuan 9654	China	KU360396	KU360404
*Gloeoporus hainanensis*	Dai 15253	China	KU360402	KU360408
*Hyphodermella poroides*	Dai 12045	China	KX008367	KX011852
*Irpex oreophilus*	Niemelä 7691	Finland	JN710548	JN710548
*Junghuhnia austrosinensis*	Dai 17540	China	**MN871755**	**MN877768**
*Junghuhnia austrosinensis*	Dai 17679	China	**MN871756**	**MN877769**
*Junghuhnia autumnale*	Spirin 2957	Russia	JN710549	JN710549
*Junghuhnia collabens*	KH Larsson 11848	Sweden	JN710552	JN710552
*Junghuhnia crustacea*	Miettinen 13852	Indonesia	JN710553	JN710553
*Junghuhnia crustacea*	Miettinen 2954	Indonesia	JN710554	JN710554
*Junghuhnia crustacea*	Dai 19138	China	**MN871757**	**MN877770**
*Junghuhnia fimbriatella*	Miettinen 2091	Russia	JN710555	JN710555
*Junghuhnia japonica*	Nuñez 1065	Japan	JN710556	JN710556
*Junghuhnia lacera*	Niemelä 8246	Finland	JN710557	JN710557
*Junghuhnia luteoalba*	KH Larsson 13238b	Estonia	JN710558	JN710558
*Junghuhnia micropora*	Spirin 2652	Russia	JN710559	JN710559
*Junghuhnia nandinae*	Dai 21107	China	**MN833677**	**MN833679**
*Junghuhnia nandinae*	Dai 21108	China	**MN833678**	**MN833680**
*Junghuhnia nitida*	KH Larsson 11903	Sweden	JN710560	JN710560
*Junghuhnia pseudozilingiana*	M Kulju 1004	Finland	JN710561	JN710561
*Junghuhnia rhinocephala*	Miettinen X460	Australia	JN710562	JN710562
*Junghuhnia* sp.	Miettinen 10026	China	JN710551	JN710551
*Junghuhnia subcollabens*	Dai 19344	China	**MN871758**	**MN877771**
*Junghuhnia subcollabens*	Dai 19345	China	**MN871759**	**MN877772**
*Mycoacia cf. columellifera*	K Hjortstam 18286	Sweden	JN710572	JN710572
*Nigroporus vinosus*	B Seitzman 2008-100	USA	JN710575	JN710575
*Skeletocutis amorpha*	Miettinen 11038	Finland	FN907913	FN907913
*Skeletocutis yunnanensis*	Dai 15709	China	KU950434	KU950436
*Skeletocutis odora*	L 13763sp	Canada	KY948830	KY948893
*Steccherinum aridum*	Bureid 110510	Norway	JN710583	JN710583
*Steccherinum bourdotii*	Saarenoksa 10195	Finland	JN710584	JN710584
Steccherinum cf. ciliolatum	Ryvarden 47033	Estonia	JN710585	JN710585
*Steccherinum fimbriatum*	KH Larsson 11905	Sweden	JN710530	JN710530
*Steccherinum litschaueri*	Spirin 2189	Russia	JN710587	JN710587
*Steccherinum murashkinskyi*	Spirin 2367	Russia	JN710588	JN710588
*Steccherinum ochraceum*	KH Larsson 11902	Sweden	JN710590	JN710590
*Steccherinum robustius*	GB 1195	Sweden	JN710591	JN710591
*Steccherinum straminellum*	KH Larsson 13849	France	JN710597	JN710597
*Steccherinum tenue*	KH Larsson 12316	United States	JN710598	JN710598
*Steccherinum tenuispinum*	Miettinen 8065	Finland	JN710599	JN710599
*Steccherinum tenuispinum*	Spirin 2116	Russia	JN710600	JN710600
*Trametopsis brasiliensis*	Meijer et al. 3637	Brazil	JN710510	JN710510

New sequences are shown in bold.

## Results

### Phylogenetic analysis

**Figure 1.**
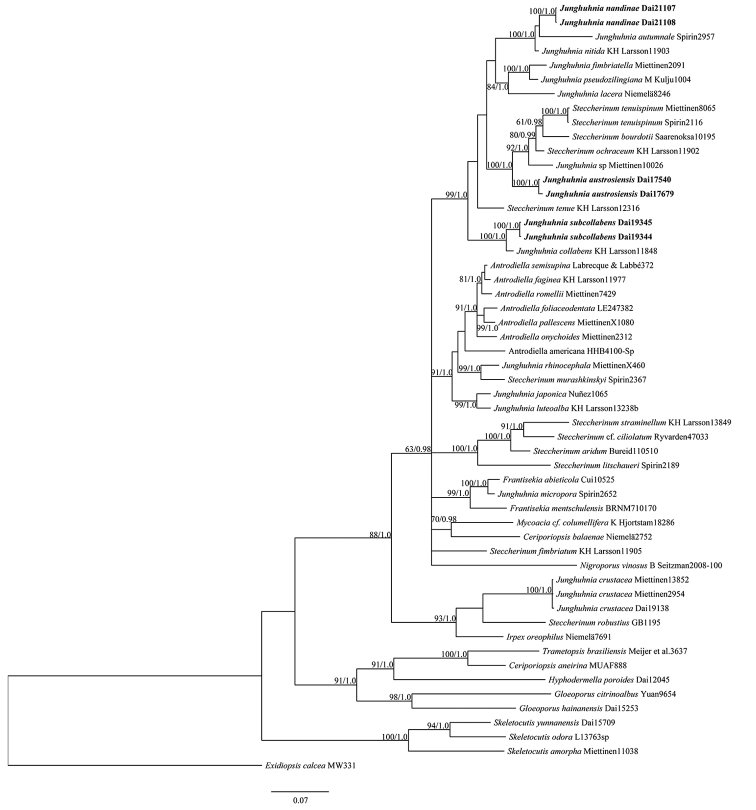
The phylogeny of three new species illustrated by Bayesian Inference tree and other taxa according to the combined ITS+nLSU dataset. Labelling of branches is done with BPP (Bayesian posterior probabilities) = 0.95 and Maximum Likelihood (ML) bootstrap greater than 50% (BS). New species are in bold.

The dataset included 54 fungal collections representing 48 species. The best model for the dataset estimated and applied in the BI was GTR+I+G. BI resulted in a similar topology with an average standard deviation of split frequencies = 0.006554 to ML analysis, and thus only the BI tree was provided. Both BPPs (≥ 0.95) and BS values (≥ 50 %) are mentioned at the nodes (Fig. 1). The three new species formed three independent lineages with robust support (BS, 100%; BPP, 1.00).

### Taxonomy

#### Junghuhnia austrosinensis

Taxon classificationFungiPolyporalesMeruliaceae

F. Wu, P. Du & X.M. Tian sp. nov.

15F5FDA1-02C4-5EB3-8EBF-3082EEB078D2

834502

Figures 2 , 3

##### Etymology.

Refers to the species being collected in the south of China.

##### Basidiomata.

Annual, resupinate, soft corky, without odour or taste when fresh, corky when dried, 7 cm length, 4 cm width and 0.4 mm thick at centre. Pore surface white when fresh, cream to buff-yellow when dried; margin distinct, white and nearly 1 mm width; pores round to angular, 9–11 per mm; dissepiments thin, entire. Subiculum cream, paler than tubes, corky when dried, nearly 0.1 mm thick. Tubes concolorous with pore surface, corky, nearly 0.3 mm length.

**Figure 2.**
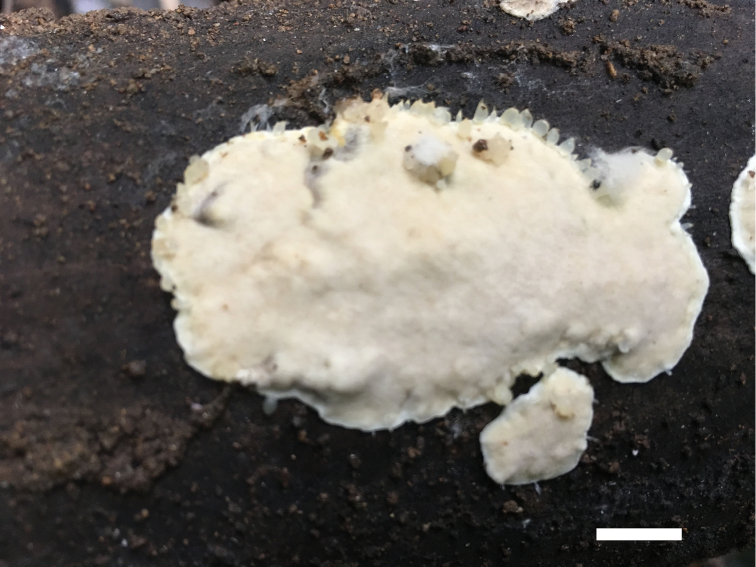
Basidiomata of *Junghuhnia austrosinensis* (holotype Dai 17540). Scale bar: 10 mm.

##### Hyphal system.

Hyphal system dimitic; generative hyphae with clamp connections, skeletal hyphae IKI–, CB+; tissue unchanged in KOH.

##### Subiculum.

Dominated by skeletal hyphae; generative hyphae hyaline, thin- to fairly thick walled, rarely branched, 2–3.5 µm in diam.; skeletal hyphae thick-walled with a wide to narrow lumen, flexuous, unbranched, gelatinised, interwoven, 3–4 µm in diam.

##### Tubes.

Trama dominated by skeletal hyphae; generative hyphae hyaline, thin- to fairly thick walled, rarely branched, 2–3 µm in diam.; skeletal hyphae thick-walled with a wide to narrow lumen, unbranched, more or less straight, subparallel amongst the tube, 2.5–3.8 µm in diam. Skeletocystidia clavate, thick-walled, originated from trama, apex covered with crystals, embedded amongst trama and dissepiments or projecting into hymenium, 30–40 × 6–8 µm; smaller skeletocystidia clavate, thick-walled, 14–18 × 5–6 µm. Basidia barrel-shaped, bearing four sterigmata and a basal clamp connection, 7–8 × 4–4.5 µm; basidioles in shape similar to basidia, but smaller.

##### Spores.

Basidiospores smooth, ellipsoid, thin-walled, hyaline, IKI–, CB–, (2.4–)2.5–3(–3.1) × (1.6–)1.7–2(–2.1) µm, W = 1.83 µm, L = 2.83 µm, Q = 1.51 (n = 30/1).

##### Materials examined.

China, Yunnan Province, Jinghong, Virgin Forest Park, on fallen bamboo, 17.VI.2017 Dai 17540 (holotype, BJFC025072, isotype in IFP). Hainan Province, Wuzhishan County, Wuzhishan Forest Park, on fallen angiosperm branch, 9.IX.2019 Dai 17679 (paratype, BJFC025211).

**Figure 3.**
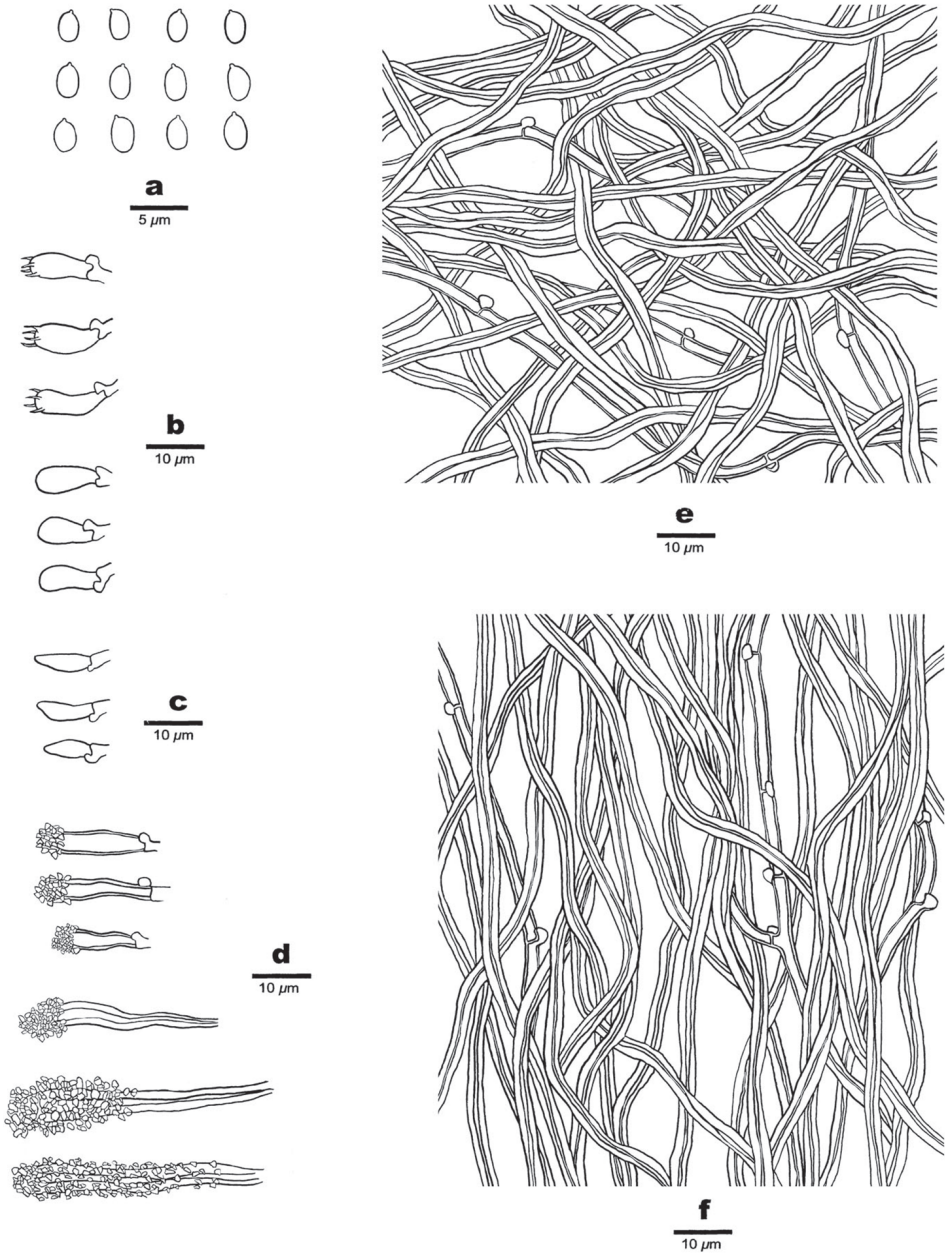
Microscopic assessment of *Junghuhnia austrosinensis* structures (drawn from Dai 17540) **a** basidiospores **b** basidia and basidioles **c** cystidioles **d** two kinds of skeletocystidia **e** hyphae from subiculum **f** hyphae from trama.

#### Junghuhnia nandinae

Taxon classificationFungiPolyporalesMeruliaceae

F. Wu, P. Du & X.M. Tian sp. nov.

DA9EFE51-5E70-5434-853E-3B12CE8362D8

833784

Figures 4 , 5

##### Etymology.

Refers to the species growing on *Nandina domestica*.

##### Basidiomata.

Annual, resupinate, coriaceous, without odour or taste when fresh, hard corky when dried, 30 cm length, 3 cm width and 1 mm thick. Pore surface flesh-pink when fresh, pink to salmon when dried; margin distinct, white and nearly 3 mm width; pores round to angular, 6–8 per mm; dissepiments thin, entire. Subiculum buff, paler than tubes, corky when dried, nearly 0.5 mm thick. Tubes concolorous with pore surface, corky, nearly 0.5 mm length.

**Figure 4.**
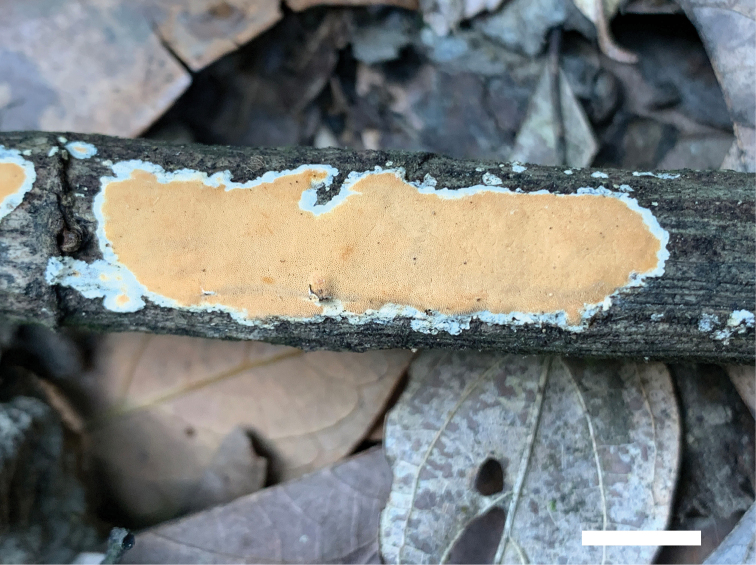
Basidiomata of *Junghuhnia nandinae* (holotype Dai 21107). Scale bar: 8 cm.

##### Hyphal system.

Hyphal system dimitic; generative hyphae with clamp connections, skeletal hyphae IKI–, CB+; tissue unchanged in KOH.

##### Subiculum.

Dominated by skeletal hyphae; generative hyphae hyaline, thin-walled, unbranched, 2–3 µm in diam.; skeletal hyphae thick-walled to subsolid, flexuous, unbranched, gelatinised, interwoven, 2.5–4 µm in diam.

##### Tubes.

Trama dominated by skeletal hyphae; generative hyphae hyaline, thin-walled, rarely branched, 2–3 µm in diam.; skeletal hyphae thick-walled to subsolid, unbranched, flexuous, more or less gelatinised, interwoven, 2.5–3.5 µm in diam. Skeletocystidia clavate, thick-walled, originated from trama, apex covered with crystals, embedded amongst trama and dissepiments or projecting into hymenium, 22–45 × 6–8 µm. Basidia clavate, bearing four sterigmata and a basal clamp connection, 8–11 × 4–4.6 µm; basidioles in shape similar to basidia, but smaller.

##### Spores.

Basidiospores ellipsoid, hyaline, thin-walled, smooth, IKI–, CB–, (2.5–)2.6–3.2(–3.3) × (1.6–)1.8–2(–2.1) µm, L = 2.97 µm, W = 1.92 µm, Q = 1.54 (n = 60/2).

##### Materials examined.

China, Chongqing, Nanchuan County, Jinfoshan Forest Park, on dead tree of *Nandina domestica*, 1.XI.2019 Dai 21107 (holotype in BJFC, isotype in IFP) and Dai 21108 (paratype in BJFC).

**Figure 5.**
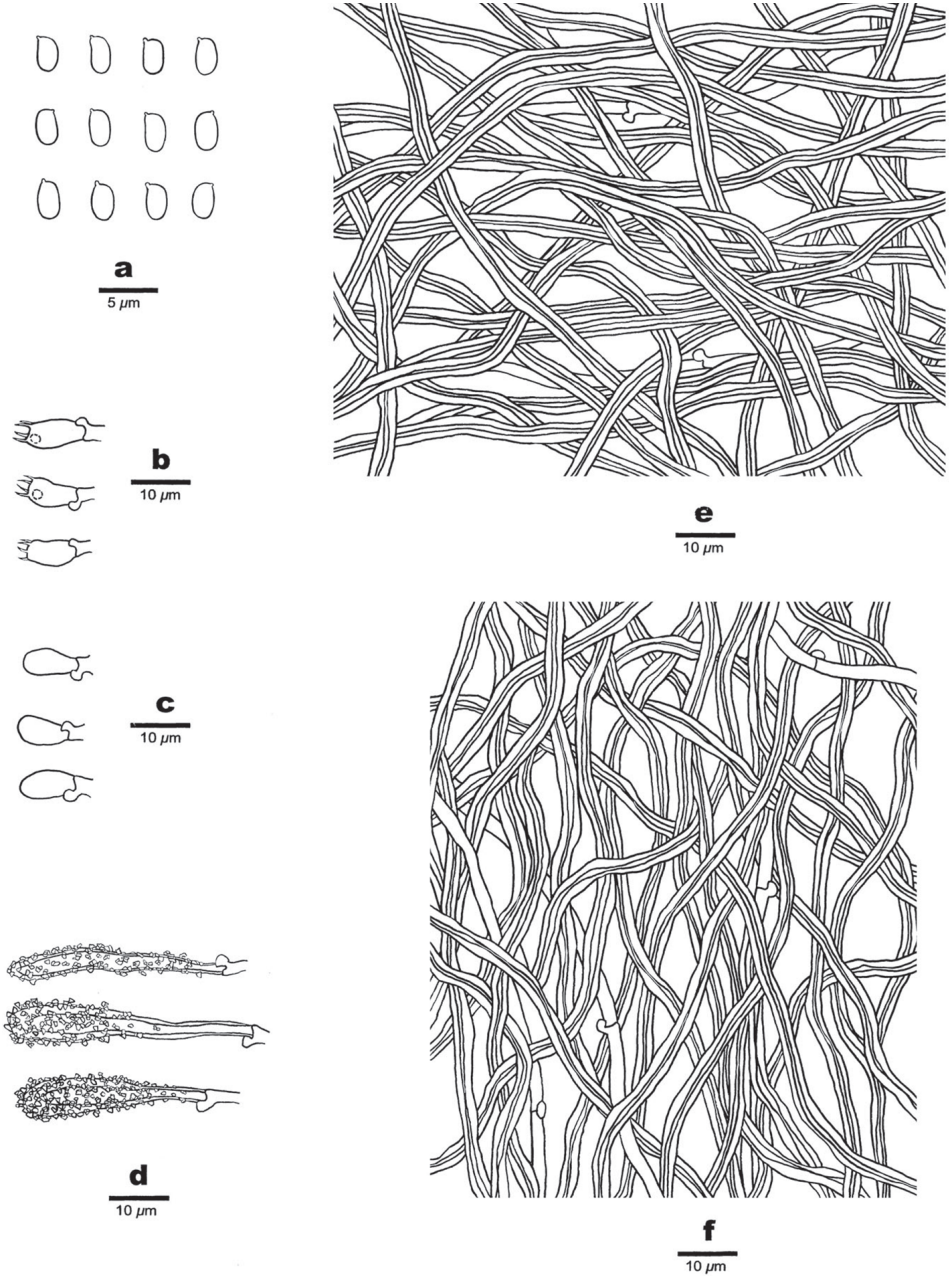
Microscopic assessment of *Junghuhnia nandinae* structures (holotype Dai 21107) **a** basidiospores **b** basidia **c** basidioles **d** skeletocystidia **e** hyphae from subiculum **f** hyphae from trama.

#### Junghuhnia subcollabens

Taxon classificationFungiPolyporalesMeruliaceae

F. Wu, P. Du & X.M. Tian sp. nov.

CD3A9B17-D75D-55B0-AEE4-F8524572E6BC

834505

Figures 6 , 7

##### Etymology.

Refers to the species similar to *J. collabens*.

##### Basidiomata.

Annual, resupinate, coriaceous, without odour or taste when fresh, hard corky when dried, 8 cm length, 3 cm width and 1.5 mm thick. Pore surface pale salmon when fresh, brownish-vinaceous when dried; margin indistinct to almost lacking; pores round to angular, 10–12 per mm; dissepiments thin to fairly thick, entire. Subiculum vinaceous, darker than pores, hard corky when dried, nearly 0.3 mm thick. Tubes vinaceous, distinctly darker than pore surface, rigid, nearly 1.2 mm length.

**Figure 6.**
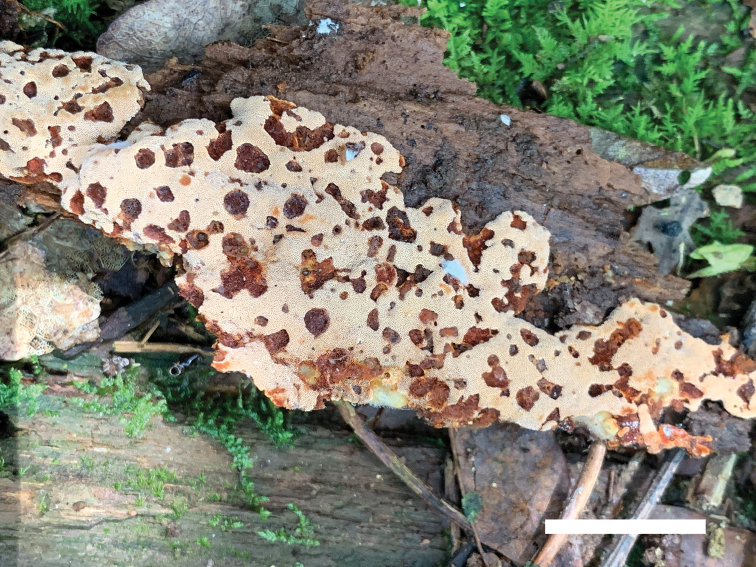
Basidiomata of *Junghuhnia subcollabens* (holotype Dai 19345). Bar: 10 mm.

##### Hyphal system.

Hyphal system dimitic; generative hyphae with clamp connections and simple septa, skeletal hyphae IKI–, CB+; tissue unchanged in KOH.

##### Subiculum.

Dominated by skeletal hyphae; generative hyphae hyaline, thin- to fairly thick-walled, frequently branched, 2.5–3 µm in diam.; skeletal hyphae thick-walled with a wide to narrow lumen, flexuous, occasionally branched, more or less gelatinised, interwoven, 2–4 µm in diam.

##### Tubes.

Trama dominated by skeletal hyphae; generative hyphae hyaline, thin- to fairly thick-walled, frequently branched, with both simple septa and clamp connections, simple septa especially common at dissepiment edge, 2–3.2 µm in diam.; skeletal hyphae thick-walled with a wide to narrow lumen, rarely branched, flexuous, more or less gelatinised, interwoven, 2.5–3.5 µm in diam. Skeletocystidia clavate, thick-walled, originated from trama, apex covered with crystals, embedded amongst trama and dissepiments or projecting into hymenium, 35–50 × 6–9 µm. Fusoid cystidioles present, 8–14 × 3.5–2.5 µm; basidia clavate, bearing four sterigmata and a basal clamp connection, 10–12 × 4–5 µm; basidioles in shape similar to basidia, but smaller.

##### Spores.

Basidiospores mostly lunate, hyaline, thin-walled, smooth, sometimes with one or two small guttules, IKI–, CB–, (2.8–)2.9–3.4(–3.5) × (1.5–)1.6–1.8(–1.9) µm, L = 3.12 µm, W = 1.67 µm, Q = 1.87 (n = 30/1).

##### Materials examined.

China, Yunnan Province, Yongping County, Baitaishan Forest Park, on rotten angiosperm wood, 7.XI.2018 Dai 19345 (holotype, BJFC027813, isotype in IFP) and Dai 19344 (paratype, BJFC027812).

**Figure 7.**
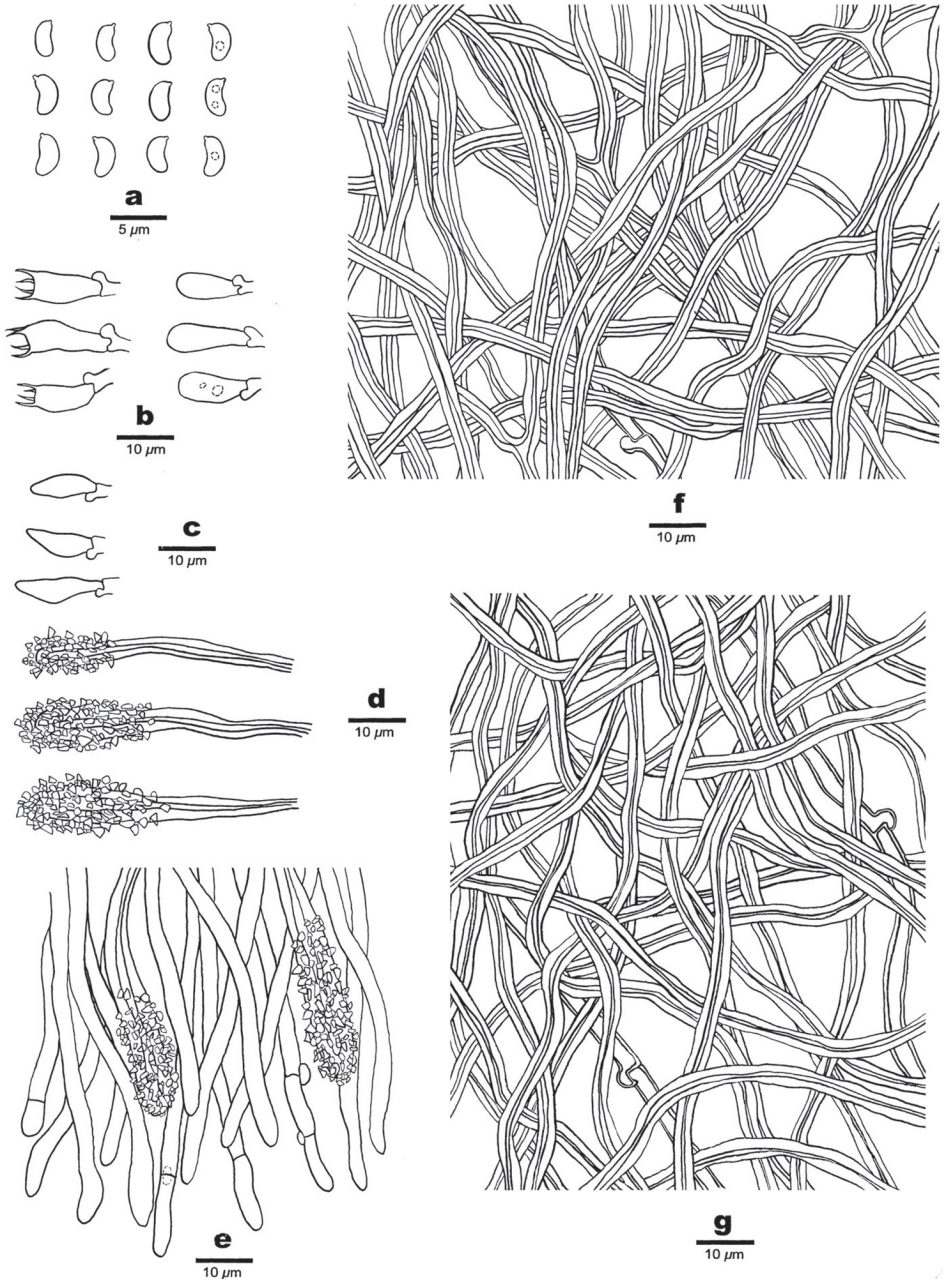
Microscopic structures of *Junghuhnia subcollabens* (holotype Dai 19345) **a** basidiospores **b** basidia and basidioles **c** cystidioles **d** skeletocystidia **e** hyphae and skeletocystidia at dissepiment **f** hyphae from subiculum **g** hyphae from trama.

## Discussion

*Junghuhnia*, *Antrodiella* and *Steccherinum* are phylogenetically related and they belong to the family of Steccherinaceae Parmasto in Polyporales (Yuan 2014; Miettinen and Ryvarden 2016; Justo et al. 2017). Our phylogeny also shows similar relationships amongst the species in the three genera (Fig. 1). Morphologically, *Junghuhnia* is distinguished from the other two genera by its poroid hymenophore and skeletocystidia. Based on phylogenetic analyses, several genera of wood-inhabiting fungi include species with lamellate, poroid and hydnaceous hymenophore at the same time (He and Dai 2012; Cui et al. 2019), but we still keep the traditional concepts for the three genera because their limited taxa were analysed according to morphology and phylogeny.

*Junghuhnia austrosinensis* is related to *Steccherinum bourdotii* Saliba & A. David, *S. ochraceum* (Pers. ex J.F. Gmel.) Gray, *S. tenuispinum* Spirin, Zmitr. & Malysheva and *Junghuhnia* sp. Miettinen 10026 (Fig. 1), but these three *Steccherinum* species have odontioid to hydnoid hymenophore and lack hymenial cystidia (Eriksson et al. 1984; Saliba et al. 1988; Spirin et al. 2007a). *Junghuhnia* sp. Miettinen 10026 was mentioned as Junghuhnia cf. semipileata (Miettinen et al. 2012), but we did not find the taxon of *Junghuhnia semipileata* (http://www.indexfungorum.org/names/Names.asp; http://www.mycobank.org/Biolomics.aspx?Table=Mycobank&Page=200&ViewMode=Basic). So far, *Skeletocutis semipileata* (Peck) Miettinen & A. Korhonen is the sole taxon with semipileata as epithet, it lacks skeletocystidia and has cylindrical basidiospores 2.8–3.1 × 0.4–0.6 µm (Korhonen et al. 2018).

*Junghuhnia minuta* I. Lindblad & Ryvarden, *J. neotropica* I. Lindblad & Ryvarden, and *J. austrosinensis* share similar pores (8–12 per mm). However, *J. minuta* has pileate basidiomata that are roughly subglobose to ellipsoid basidiospores (2–2.5 × 2.5–3 µm, Lindblad and Ryvarden 1999) and *J. neotropica* has smooth cystidia (Lindblad and Ryvarden 1999). *Junghuhnia rhizomorpha* H. S. Yuan & Y. C. Dai resembles *J. austrosinensis* by having resupinate basidiomata and almost the same size pores (8–10 per mm), but the former has rhizomorphs, wider basidiospores and lacks hymenial cystidia (2.7–3 × 1.9–2.1 µm, Yuan and Dai 2008).

Phylogenetically, *Junghuhnia nandinae* is closely related to *J. nitida* (Pers.) Ryvarden and *J. autumnale* Spirin, Zmitr. & Malysheva (Fig. 1), but *J. nitida* has larger basidiospores (4–4.5 × 2.4–2.9 µm, Niemelä 2016) and *J. autumnale* differs from *J. nandinae* by pileate basidiomata, larger pores (5–7 per mm) and larger basidiospores (3.1–4.1 × 2.1–3 µm, Spirin et al. 2007b). Morphologically, *J. nandinae* resembles *J. collabens* (Fr.) Ryvarden in terms of salmon coloured pores, but the latter has cylindrical to suballantoid basidiospores (3.2–3.6 × 1.4–1.7 µm) and grows on gymnosperm wood in temperate and boreal forests (Niemelä 2016), while *J. nandinae* has ellipsoid basidiospores and is so far found in subtropical areas in China. The following names were treated as synonyms of *J. nitida*: *Poria fulgens* Rostk., *Polyporus euporus* P. Karst., *Physisporus vitellinulus* P. Karst. and *Chaetoporus tenuis* P. Karst. (http://www.indexfungorum.org/Names/Names.asp). All these taxa were originally described from Europe and they most probably represent a single species of *J. nitida*.

*Junghuhnia subcollabens* is phylogenetically closely related to *J. collabens* (Fig. 1) and both species share salmon pore surfaces, but *J. collabens* differs from *J. subcollabens* by larger pores (6–8 per mm), cylindrical to suballantoid basidiospores (3.2–3.6 × 1.4–1.7 µm), lacking simple septa on generative hyphae and growing on gymnosperm wood in temperate and boreal forests (Niemelä 2016), while *J. subcollabens* has smaller pores (10–12 per mm), lunate basidiospores (2.9–3.4 × 1.6–1.8 µm), simple septa on generative hyphae and growing on angiosperm wood in warm temperate forests of southwest China.

Three new species of *Junghuhnia* are described from Southern China in the present paper. Although extensive surveys on wood-decaying fungi in Southern China were carried out, and more than 3000 specimens were collected with 132 new polypore (Dai 2010; Zhao et al. 2015; Chen et al. 2020; Wu et al. 2020), it is expected that more new taxa will be found after additional investigations based on careful morphological examinations and phylogenetic analyses because of the rich woody plant species in subtropical and tropical China.

## Supplementary Material

XML Treatment for Junghuhnia austrosinensis

XML Treatment for Junghuhnia nandinae

XML Treatment for Junghuhnia subcollabens
